# Coronary Anatomy in Congenital Heart Disease: The Important Contributions of Professor Dr. Adriana Gittenberger-de Groot

**DOI:** 10.3390/jcdd8030027

**Published:** 2021-03-09

**Authors:** Mark Hazekamp

**Affiliations:** Department of Cardiothoracic Surgery, D6-26, Leiden University Medical Center, Albinusdreef 2, 2333ZA Leiden, The Netherlands; M.G.Hazekamp@lumc.nl

**Keywords:** transposition great arteries coronary anatomy

## Abstract

The contributions of Professor Dr. Adriana Gittenberger-de Groot in relation to coronary artery development and classification are described from the viewpoint of a pediatric cardiac surgeon.

Professor Dr. Adriana Gittenberger-de Groot has contributed in many ways to the vast field of congenital heart disease. Her research in this area comprises many different topics and is too extensive to summarize in one paper.

As a surgeon who is specialized in congenital heart disease, I will focus on one field where Adriana Gittenberger-de Groot has made contributions that have been of especially great value to me and to my fellow pediatric cardiac surgeons. I here refer to her work on coronary development and anatomy in congenital heart disease. This has ultimately resulted in the so-called Leiden Convention, a coronary coding system where the origin and location of the coronary arteries in hearts with congenital heart disease, such as transposition of the great arteries (TGA), is described in a way that is both simple and unequivocal. The strength of the system lies in that it is independent of the relative anatomical position of the great arteries, which can vary in different forms of congenital heart disease. This classification greatly facilitates communication between pediatric cardiologists and pediatric heart surgeons when they speak on how the coronary arteries are distributed in a patient with TGA. Its simplicity and the fact that it is always applicable underlines the beauty of the Leiden Convention coronary coding system [1].

The first arterial switch operation in Leiden was performed in 1977, shortly after Jatene had done his first successful arterial switch in Sao Paulo in 1975 [2]. The most critical part of this surgical procedure was (and still is) the translocation of the coronary arteries from their original position in the aorta to their new place in the pulmonary truncus, which will function as the new aorta after the arterial switch operation. Variations in the origin and course of the coronary arteries are common, especially when TGA is combined with a ventricular septal defect. As specific variations significantly increase the risk of the arterial switch operation, it is of great importance to know the coronary anatomy before surgery is started. A classification that is both simple and comprehensive was therefore desperately needed with the evolution of this surgical technique.

The classification that Adriana Gittenberger-de Groot proposed was independent of the spatial relation between the great arteries and was based on the observation that the coronary arteries always arise from the facing sinuses. In short, an observer is positioned in the non-facing sinus of the aorta and looks towards the pulmonary artery. The sinus on the right-hand side is called “sinus 1”, and the sinus on the left-hand side is called “sinus 2”. Then the three main coronary arteries are each named with the observer looking in a counterclockwise fashion, starting in sinus 1. Thus, the most common anatomy in TGA will be 1LCx-2R, where 1 and 2 denote the sinuses, L is left anterior descending artery, Cx is the circumflex artery, and R stands for the right coronary artery. The nomenclature was elegantly described by the Leiden pediatric cardiac surgeon Jan Quaegebeur after studying many clinical and autopsy cases of TGA [3]. The Leiden Convention is for all reasons mentioned above more understandable than other classifications, such as Yacoub type A–D [4,5].

The embryological aspects of coronary artery development have always been a topic of interest for Prof. Dr. Gittenberger-de Groot, as demonstrated by several studies that have led to the refutation of previous theories [6,7]. This work has shown that coronary arteries do not grow from the aorta towards the periphery, but on the contrary, coronary arteries grow toward the aorta. This provides explanations for coronary artery variations in TGA, in common arterial trunk but for also the occurrence of aberrant coronary arteries that arise from the pulmonary artery [8,9]. All this is of importance to the congenital cardiac surgeon.

Many years later, the same topic resurfaced when Adriana Gittenberger-de Groot started looking at coronary artery anatomy in relation to bicuspid aortic valve [10,11]. Ultimately, this led to a modification of the Leiden Convention that encompasses coronary arterial origin and branching not only in hearts with TGA or double outlet right ventricle but also in hearts with normally related great arteries, hearts with different types of bicuspid aortic valve, or hearts that have coronary arteries that arise from another aortic sinus. In bicuspid aortic valve, the Leiden Convention is applicable in 99% of cases (not in the very rare cases where there is no raphe). Furthermore, the Leiden Convention can be used to determine which leaflets are fused. The newly modified Leiden Convention stands for a simple coronary coding system that is broadly applicable (Figure 1).

In summary, the contributions of Adriana Gittenberger-de Groot have helped to advance new theories and change the way of thinking in the field of coronary artery development and coronary artery variations in TGA and bicuspid aortic valve. The Leiden Convention and the more recently modified Leiden Convention provide a simple, unambiguous coding system of coronary artery origin and branching that is of great help to surgeons who dedicate their efforts to the treatment of congenital heart disease.

## Figures and Tables

**Figure 1:**
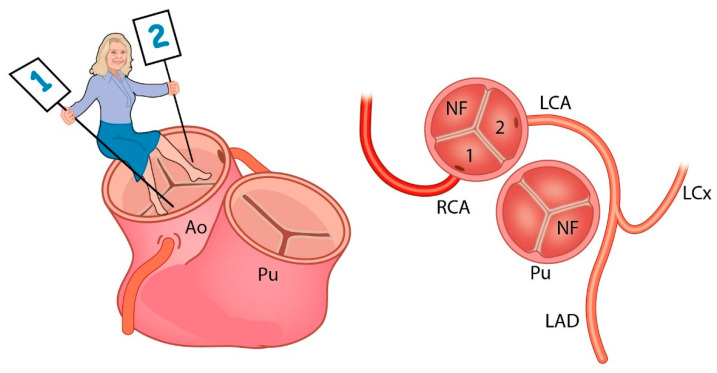
Ao: aorta, Pu: Pulmonary artery, NF: Non Facing sinus, RCA: Right Coronary Artery, LCA: Left Coronary Artery, LAD: Left Anterior Descending artery, LCx: Left Circumflex artery. The observer sits in the non-facing sinus of the aorta and looks towards the facing sinuses. Sinus 1 is on her right-hand side, while sinus 2 is on her left-hand side. The coronary arteries are described in a counterclockwise fashion: 1RCA-2LAD LCx. (Adapted with permission from: Gittenberger de Groot et al. J Thorac Cardiovasc Surg. 2018 Dec;156(6):2260–2269).
